# Redox Regulation of PTEN by Peroxiredoxins

**DOI:** 10.3390/antiox10020302

**Published:** 2021-02-16

**Authors:** Thang Nguyen Huu, Jiyoung Park, Ying Zhang, Iha Park, Hyun Joong Yoon, Hyun Ae Woo, Seung-Rock Lee

**Affiliations:** 1 Department of Biochemistry, Research Center for Aging and Geriatrics, Research Institute of Medical Sciences, Chonnam National University Medical School, Gwangju 501-190, Korea; huuthangsmp@gmail.com (T.N.H.); ip071@hanmail.net (I.P.); hjms0320@hanmail.net (H.J.Y.); 2 Department of Biomedical Sciences, Research Center for Aging and Geriatrics, Research Institute of Medical Sciences, Chonnam National University Medical School, Gwangju 501-190, Korea; 3 College of Pharmacy, Graduate School of Pharmaceutical Sciences, Ewha Womans University, Seoul 120-750, Korea; jypark89@ewhain.net; 4 Department of Cell Biology, School of Medicine, Jiangsu University, Zhenjiang 212013, China; zhangying4097@163.com

**Keywords:** PTEN, redox regulation, peroxiredoxins

## Abstract

Phosphatase and tensin homolog deleted on chromosome 10 (*PTEN*) is known as a tumor suppressor gene that is frequently mutated in numerous human cancers and inherited syndromes. PTEN functions as a negative regulator of PI3K/Akt signaling pathway by dephosphorylating phosphatidylinositol (3, 4, 5)-trisphosphate (PIP3) to phosphatidylinositol (4, 5)-bisphosphate (PIP2), which leads to the inhibition of cell growth, proliferation, cell survival, and protein synthesis. PTEN contains a cysteine residue in the active site that can be oxidized by peroxides, forming an intramolecular disulfide bond between Cys^124^ and Cys^71^. Redox regulation of PTEN by reactive oxygen species (ROS) plays a crucial role in cellular signaling. Peroxiredoxins (Prxs) are a superfamily of peroxidase that catalyzes reduction of peroxides and maintains redox homeostasis. Mammalian Prxs have 6 isoforms (I-VI) and can scavenge cellular peroxides. It has been demonstrated that Prx I can preserve and promote the tumor-suppressive function of PTEN by preventing oxidation of PTEN under benign oxidative stress via direct interaction. Also, Prx II-deficient cells increased PTEN oxidation and insulin sensitivity. Furthermore, Prx III has been shown to protect PTEN from oxidation induced by 15s-HpETE and 12s-HpETE, these are potent inflammatory and pro-oxidant mediators. Understanding the tight connection between PTEN and Prxs is important for providing novel therapies. Herein, we summarized recent studies focusing on the relationship of Prxs and the redox regulation of PTEN.

## 1. Introduction

**Figure 1:**
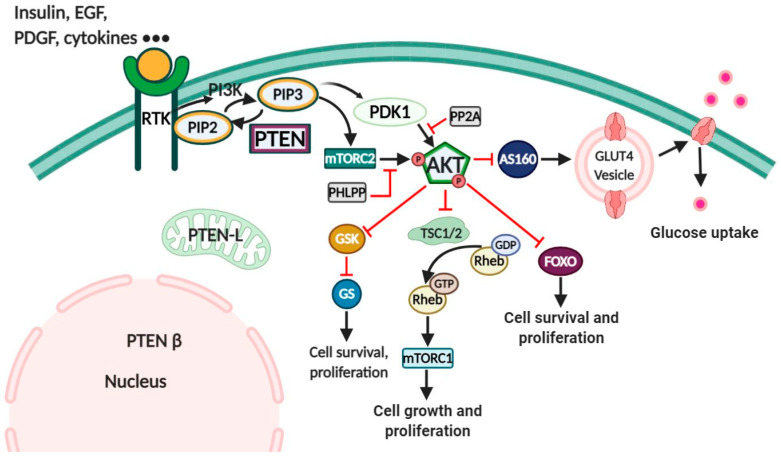
PTEN and PI3K/Akt signaling pathway. Triggering of PI3K by ligands-activated receptors (RTK, GPCR) leads to PIP3 generation. Akt is then activated through phosphorylation by mTORC2 and PDK1, which triggers the downstream signaling through a series of phosphorylation reactions. Activated Akt promotes translation of glucose transporter 4 (GLUT4) through direct inhibition of AS160 (Akt substrate of 160 kDa), which leads to the increase of glucose uptake. Furthermore, through phosphorylation, Akt inactivates forkhead box protein O (FOXO), tuberous sclerosis complex 1/2 (TSC1/2), and glycogen synthase kinase 3 (GSK3), which increases cell survival, cell growth, and proliferation [23]. PTEN dephosphorylates PIP3 to PIP2 and reduces PIP3 accumulation. Black arrows (activating), red arrows (blocking).

The phosphoinositide-3-kinase/protein kinase B (PI3K/Akt) pathway is the critical intracellular signaling in controlling a variety of cellular processes [1]. PI3Ks are intracellular lipid kinases that are conserved from yeast to mammals. It catalyzes the phosphorylation reaction of phosphatidylinositol (PI) at hydroxyl groups to form phosphoinositides [2]. There are three classes of PI3Ks: class I, II, and III, depending on the structure and substrate specificity. Among three classes, class I PI3K has the primary function of inducing the accumulation of PIP3 by PIP2 phosphorylation [2,3,4]. Class I PI3K is also classified into 2 subgroups, called class IA and class IB, depending on the signaling receptors that activate them. While the activation of class IA is induced by growth factor receptor tyrosine kinases (RTKs), the activation of class IB is induced by G protein-coupled receptors (GPCRs) [2,3,4]. All the members of class I have the heterodimeric structure that contains a regulatory subunit and a catalytic subunit: class IA with a p85 regulatory subunit and a p110 catalytic subunit, class IB with two regulatory subunits (p101, p84/p87^PIKAP^) and a p110γ catalytic subunit [2,3,4]. Triggering of the serine/threonine-specific protein kinase Akt after PIP3 generation leads to the activation of an abundance of the downstream targets [5], which is depicted in Figure 1. Akt PH domain binds to PIP3 after recruitment to the membrane [6] and Akt activation is fully completed when it is phosphorylated by both phosphoinositide-dependent kinase 1 (PDK1) at Threonine 308 in the activation loop [7,8] and the rapamycin complex-2 (mTORC2) at Serine 473 [9]. Other kinases, such as mitogen-activated protein kinase-activated kinase 2 (MAPKAPK-2), integrin-linked kinase, and PKB itself, are also known to induce Akt activation [10]. On the contrary, Akt activity can be inhibited by dephosphorylation via protein phosphatase 2A (PP2A) and PH domain leucine-rich repeat protein phosphatases (PHLPP) [11,12].

ROS are predominantly generated as by-products during various physiological processes and have both advantageous and disadvantageous effects in mammals [13]. At low concentrations, ROS have positive effects by means of increasing cellular antioxidative defense systems which enables cells to survive against higher levels of oxidative stress. ROS are also required for the regulation of intracellular signaling, which affects various cellular processes, including proliferation, cell survival, and other important events [14,15,16]. The signaling messenger function is exerted by triggering the reversible oxidation of the regulatory proteins’ cysteines. As the cells lack enzymes to remove hydroxyl radicals, further reactions can happen that leads to irreversibe oxidation and degradation of protein functions [17,18], which contributes to various diseases, such as diabetes, obesity, and cancer [19,20].

PTEN has an antagonizing function in the PI3K/Akt pathway by inhibiting the downstream signaling molecules via dephosphorylation of PIP3 to PIP2. Because PTEN possesses a cysteine in the phosphatase domain, it becomes a target of ROS, especially hydrogen peroxide (H_2_O_2_), which can oxidize and inactivate PTEN phosphatase activity, as mentioned by numerous studies [21,22]. In addition to thioredoxin (Trx), a system that can use its active cysteine residues for reducing oxidized proteins (contains disulfide bonds), Prxs are members of the thiol-dependent antioxidant family that acts as a scavenger of cytosolic or mitochondrial peroxides such as H_2_O_2_. Prxs undergo modifications in their active site, especially the cysteine residues, which enables the protective function through protein-protein interactions, or subcellular protein targeting. The spotlight of this review is to show the connection between Prxs and redox regulation of PTEN.

## 2. Characterization of PTEN

*PTEN*, located at chromosome 10q23, was firstly identified in 1997. *PTEN* mutations were found in an assortment of human diseases, for example, brain, breast, and prostate tumors. The report has shown that mutated *PTEN* accounted for 31% of glioblastoma cell lines and xenografts, 100% of prostate cancer cell lines, 6% of breast cancer cell lines and xenografts, and 17% of primary glioblastomas [24].

*PTEN* encodes a protein of 403 amino acids that consists of five essential domains: an N-terminal PIP2-binding domain (PBD), a phosphatase domain, a C2 domain, a carboxy-terminal tail domain, and a PDZ-binding motif [25,26]. The PBD domain localizes at the N-terminal of PTEN and is significant for PTEN catalytic function and membrane localization [27,28]. The phosphatase domain (the catalytic domain) comprises a conserved motif, called HCXXGXXR, which is found as a homology of the catalytic sequence in protein tyrosine phosphatases (PTPs) [29]. When compared to PTPs, the PTEN phosphatase sequence is similar to dual-specificity protein phosphatases (DUSPs) [24,30]. Furthermore, the N-terminal first 190 amino acids are also homologous to the actin-binding protein tensin 1 (TNS1) and auxilin, which is not linked with the PTEN catalytic function [24,30]. The C2 domain (amino acids 186–351) can bind phospholipid membrane independent of calcium because it lacks the canonical Ca^2+^ chelating residues in vitro, which makes PTEN inhibit cell migration [31]. The C-terminal domain (amino acid 353–403) of PTEN consists of 2 PEST sequences, including phosphorylated serine-threonine spots and a PDZ (PSD-95, DLG, ZO-1) binding motif [32]. This domain was also found to be mutated in tumors. The phosphorylated serine-threonine spots contain Ser^362^, Thr^366^, Ser^370^, Ser^380^, Thr^382^, Thr^383^, and Ser^385^ residues. There are some kinases inducing C-terminal domain phosphorylation: casein kinase 2 (CK2), GSK3β, LKB1, and MAST [33]. When the C-terminal domain is phosphorylated, PTEN stability is increased while PTEN phosphatase activity is decreased [34]. The PTEN PDZ-binding motif is also deleted in some tumors. This motif participates in the inhibition of cell migration and protein synthesis; it also stabilizes PTEN at the plasma membrane [2,35,36,37].

Several recent studies have demonstrated that PTEN has two isoforms: PTEN-Long (PTEN-L) and PTEN-β [32]. The translation of PTEN-L starts from 519 base pairs upstream compared to the initiation site of canonical PTEN. Therefore, PTEN-L was found to have additional 173 amino acids than canonical PTEN. PTEN-L is considered as a PTEN variation. PTEN-L can be secreted from cells and quickly adopted by others [38]. Furthermore, PTEN-L is found in human blood, especially plasma and serum. Because of a poly-Arginine extension in the PTEN-L structure, the PI3K/Akt inhibition is promoted both in vitro as well as in vivo. Thus, in several studies, PTEN-L was utilized as a therapy to repress tumor progression. Other findings revealed the presence of PTEN-L in mitochondria where PTEN-L and canonical PTEN interacts, leading to the augmentation of PTEN-induced putative kinase 1 (PINK1) protein that is involved in the regulation of the mitochondrial function and energy production [39]. In contrast to PTEN-L, PTEN-β is mostly restricted to the nucleus and is found to interrelate with nucleolin. PTEN-β also affects nucleolin by dephosphorylation, which participates in the negative regulation of the transcription of DNA and biogenetic ribosome [40]. Since PTEN-L and PTEN β have a huge homologous sequence with canonical PTEN, they might be regulated by the same method.

It is well-known that the major function of PTEN is a lipid phosphatase, and its main substrate is intracellular PIP3. PTEN exerts its role as a negative regulator of the proto-oncogenic PI3K/Akt pathway, which leads to the inhibition of the downstream signaling [41,42]. It has been demonstrated that PTEN can autodephosphorylate and dephosphorylate some of its substrates, for example, focal adhesion kinase 1 (FAK), cAMP-responsive element-binding protein 1 (CREB1), proto-oncogene tyrosine-protein kinase SRC, and insulin receptor substrate 1 (IRS1) [43,44,45,46,47]. PTEN also exhibits scaffold functions in both the nucleus and cytoplasm, which suppresses tumors independent of PIP3 and the PI3K/Akt axis [25,48].

The PTEN phosphatase activity can be influenced by various factors, including PTEN post-translational modifications (PTMs) and PTEN-interacting proteins. It has been shown that several PTMs, including oxidation, S-nitrosylation, and acetylation, were found to regulate PTEN. PTEN is susceptible to oxidation since it harbors a cysteine residue at the catalytic site like other PTPs. This has been illustrated in various studies [22,49,50,51]. For instance, H_2_O_2_ can induce PTEN oxidation, which inactivates PTEN phosphatase function by establishing a Cys^124^-Cys^71^ disulfide bond [22]. However, oxidized PTEN form is gradually decreased by intracellular reducing systems, which is predominantly supported by Trxs [22]. Some organic peroxides and hydroperoxides such as cumen hydroperoxide (CuHP) and *tert*-butyl hydroperoxide (*t*-BHP) have been shown as tumor promoters. When cells were stimulated with CuHP or *t*-BHP at various concentrations, PTEN was quickly oxidized and the disulfide bond was formed [49,50]. In contrast to H_2_O_2_, CuHP and *t*-BHP induced irreversible PTEN oxidation, since the Trx was also targeted to oxidation and functional inhibition via dimerization [49,50]. Furthermore, the indirect inactivation of PTEN phosphatase following oxidation is regulated by PTEN interacting proteins. For example, the Parkinson disease protein 7 (PARK7) was found to repress the PTEN phosphatase function by binding to PTEN. When the affinity between PTEN and PARK7 increases, PTEN phosphatase activity is decreased [52].

S-nitrosylation is also an important mechanism that affects the redox status of PTEN. Numerous studies have illustrated that nitric oxide (NO) is an agent inducing S-nitrosylation of PTEN, leading to the repression of both PTEN phosphatase function and the Akt downregulation [53]. It has been shown that PTEN Cys^83^ is S-nitrosylated [54]. Recently, it has been found that the impairment of PARK2 can induce the suppression of PTEN by S-nitrosylation through increase the level of NO [55]. These data suggest that the S-nitrosylation is another PTM of PTEN and could be a potential target for therapeutic purposes.

Acetylation was also found to be one of the PTMs that control PTEN activity and function. Lys^125^ and Lys^128^ residues of PTEN are modified by acetyltransferase PCAF (or KAT2B) in response to growth factor stimulation [56], which leads to the PTEN phosphatase inhibition. In addition, p300-CREB-binding protein (CBP) has been shown to acetylate PTEN at Lys^402^ in the PDZ-binding motif. Acetylation of PTEN intervenes in the interaction between PTEN and its interacting proteins [57]. Moreover, NAD-dependent protein deacetylase sirtuin 1 (SIRT1) is reported to be involved in the deacetylation of PTEN reaction. Hyperacetylation of PTEN has been demonstrated in cells that lack SIRT1 [57,58]. PTEN is translocated from the nucleus to the cytosol upon SIRT1 depletion [58], suggesting that acetylation regulates its subcellular localization.

PTEN phosphatase activity can be regulated by protein-protein interactions, which affects its stability, subcellular localization, and affinity. Several studies have shown that PIP3-dependent Rac exchanger 2 protein (PREX2) [59], shank-interacting protein-like 1 (SIPL1; or SHARPIN) [60], and α-mannosidase 2C1 (MAN2C1) [61] can directly suppress PTEN lipid phosphatase activity by acting as negative regulators of PTEN. The phosphorylated Akt levels are also enhanced by these negative regulators, further indicating that the cellular activity of Akt depends on PTEN inhibition.

## 3. Characterization of Mammalian Peroxiredoxins

Peroxiredoxins (Prxs) are members of the small peroxidase family (22–27 kDa), which participate in the reduction of H_2_O_2_, organic hydroperoxides, and peroxynitrite since they possess thiol residues in their active sites [62,63,64,65,66]. Prxs are widely discovered in the mid-1990s [67], its scavenging role was long overshadowed by other protection systems, such as catalase and glutathione peroxidase (Gpx), but upgraded kinetics estimations presently demonstrate that Prxs are also crucial enzymes for reducing cellular peroxides [13,68]. At the NH_2_-terminal site of Prxs, it has a conserved peroxidatic cysteine residue (C_P_) that is easily oxidized by peroxides [69,70]. The other additional cysteine residue found in some Prxs, called resolving Cys (C_R_), is located at the COOH-terminal site of the molecules. Based on the presence or location of the C_R_ residue, members of Prxs are divided into three groups: 2-Cys proteins, atypical 2-Cys proteins, and 1-Cys proteins [62]. There are a total of six Prxs isoforms in mammals: four 2-Cys Prx isoforms (Prx I to Prx IV), one atypical 2-Cys isoform (Prx V), and one 1-Cys Prx isoform (Prx VI) [65]. These isoforms differ in their subcellular localization and substrate preferences. In general, mammalian Prx I to Prx IV are more efficient in reducing H_2_O_2_, whereas Prx V is more effective against alkyl hydroperoxides and peroxynitrite compared to H_2_O_2_ and Prx VI is more active against alkyl hydroperoxides over other peroxides [71,72,73]. Despite each of these isoforms has a distinctive function in cellular redox protection, they all have the intracellular H_2_O_2_-reducing activity that can control the levels of intracellular H_2_O_2_ [74].

Prxs exist in a head-to-tail dimeric structure. During the reaction with H_2_O_2_, the C_P_-SH residue was transformed to cysteine sulfenic acid (C_P_-SOH). In the case of the 2-Cys Prxs, this unstable sulfenic acid forms an intermolecular disulfide bond with a C_R-_SH residue of the other molecule in the dimeric Prx structure. This disulfide bond is then reduced by the Trx system, which is firstly identified in yeast Prx (TSA) [75,76] when the crystal structure of rat Prx I was investigated and the intermolecular disulfide was found between Cys^52^ and Cys^173^ [77]. Mutant forms of Prx I to Prx IV that lack either C_P_ or C_R_ thus do not manifest Trx-dependent peroxidase activity [62]. Prx V and Prx VI have a similar initial step with 2-Cys Prxs in peroxidase cycles, which involves the oxidation of C_P_-SH (Cys^47^ for both human isoforms) into C_P_-SOH. In the case of Prx V, an intra-subunit disulfide bond forms between C_P_-SOH with Cys^151^ (C_R_-SH), and this disulfide bond is reduced by Trx [78]. Prx V dimeric structure with an intramolecular disulfide bond was determined in the Prx V crystal structure [72,79,80], which uncovered that Prx V dimers are not antiparallel and that Prx V interface-involved dimerization is different from that of Prx I to Prx IV and of Prx VI [72]. Prx VI is absent of C_R_, thus, Prx VI C_P_-SOH cannot be resolved within the dimer. A cysteine thiol (C_R_-SH) of the π isoform of glutathione S-transferase (πGST) was found to resolve the Prx VI–SOH [81,82,83]. The resulting heterodimeric disulfide (PrxVI–S–S–πGST) is reduced by two glutathione (GSH) molecules that are converted to oxidized glutathione (GSSH) [81,82,83]. Oxidized Prx VI is also reduced by dithiothreitol (DTT) but not by Trx. The Prx VI homodimer crystal structure is antiparallel to Cys^47^-SOH that was steadied by other surrounding residues [84]. Like Prx V, Prx VI functions as a monomer.

**Figure 2:**
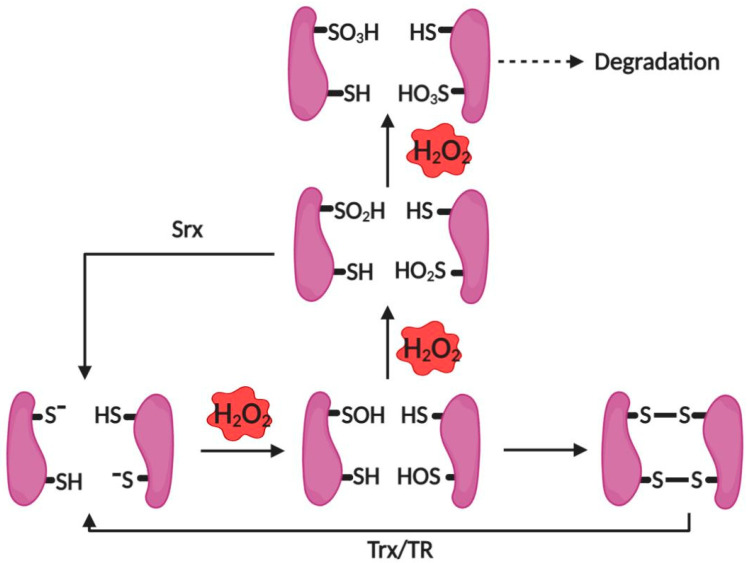
Reactions between Prx I, Trx, Srx and H_2_O_2_.

Prx I belongs to the 2-Cys Prx group, therefore, when exposed to low levels of H_2_O_2_, the sulfenic acid (C_P_-SOH) was formed at the site of Cys^51^ after C_P_-SH oxidation. The intermolecular disulfide bond is formed after interacting with Cys^172^ of the other molecule in the Prx I dimeric structure. Eventually, this disulfide bond is reduced by Trx [62,76]. Upon exposure to higher levels of H_2_O_2_, a further oxidative reaction occurs that leads to oxidation of C_P_-SOH into C_P_-SO_2_H (sulfinic acid), subsequently inactivating its peroxidase activity. This sulfinic acid can undergo the reduction into thiol by Sulfiredoxin (Srx) system [85,86], whereas further oxidation of C_P_-SO_2_H to C_P_SO_3_H (sulfonic acid) is irreversible, which degrades Prx [85]. These reactions are shown in Figure 2. It has been shown that higher molecular weight complexes were preferred when Prx I is overoxidized because of oxidative stress or heat shock stress [87]. Accompanying the structural changes, the function of Prx I also alters from peroxidase to molecular chaperone activity [88,89].

The Prx peroxidase activity is regulated by numerous PTMs, such as phosphorylation, deacetylation, and S-nitrosylation [90,91,92]. It has been observed that membrane-associated Prx I is phosphorylated on Tyr^194^ in vitro upon stimulation of various receptors (Epidermal growth factor receptor—EGFR, platelet-derived growth factor—PDGF, B-cell receptor—BCR, and T-cell receptor—TCR) in several cell lines as well as in vivo following cutaneous injury in mice, thus contribute to remaining Nox-generated H_2_O_2_ levels for intracellular messenger function [90]. Furthermore, Prx I and Prx II have known as substrates of histone deacetylase 6 (HDAC6). Acetylated Prx I and Prx II increased in cell lines that lack HDAC6 and in HDAC6-knockdown conditions [91]. The acetylated form of Prx was also proved to be more effective in H_2_O_2_ reduction, which suggests potential therapeutic targets can be developed for the increase of acetylated Prx, including HDAC6 inhibitor [91]. Prx II was S-nitrosylated by nitric oxide (NO) both in vitro in human SH-SY5Y cells (cell-based models of Parkinson’s disease-PD) and in vivo in human PD brain tissue [92]. Prx II S-nitrosylation occurred at two cysteine residues (Cys^51^ and Cys^172^), which leads to the inactivation of Prx peroxidase activity [92]. Therefore, the relationship between NO and oxidative stress in neurodegenerative disorders was demonstrated partly through Prx II S-nitrosylation [92].

## 4. Redox regulation of PTEN by Peroxiredoxins

### 4.1. Regulation of PTEN by Prx I

It has been demonstrated that, under mild oxidative stress, PTEN tumor-suppressive activity was protected by Prx I via forming the intermolecular disulfide bond [93]. At the low level of H_2_O_2_ (25 µM), the PTEN lipid phosphatase activity was completely protected by Prx I in cells, as the interaction between Prx I and PTEN was found. On the other hand, at high levels of H_2_O_2_ (500 μM), the hyperoxidation forms of Prx I can be observed with the dissociation from PTEN. As a result, at the 1:1 ratio of Prx I and PTEN, the high concentrations of H_2_O_2_ can oxidize Prx I at Cys^51^ and promote unwinding of the PTEN-binding conformation of Prx I consequently inducing their dissociation [93]. In addition, the results also showed that the 1:1 ratio of Prx I and PTEN is the most effective ratio for protecting PTEN phosphatase activity, and additional amounts of Prx I could not increase the protection, suggesting the monomeric interaction between Prx I and PTEN [93]. Mutational analysis and computational analysis suggested that Prx I interacts within the C2 domain of PTEN (amino acid 186–274) and PTEN with the N-terminal of Prx I (amino acid 1–21) and the C- terminal of Prx I (amino acid 183–199) [93].

Exposure to H_2_O_2_ for various times resulted in the formation of hyperoxidized Prxs. In Hela cells untreated with H_2_O_2_, the Prx I dimeric forms were predominantly observed since Prxs are considered as dimers in the absence of NEM [94]. The Prx I hyperoxidation levels reached a peak after 5 min of exposure and then gradually declined. Besides, the increase of oxidized PTEN levels showed similar kinetics to H_2_O_2_-induced Prx I hyperoxidation, and the transient hyperoxidation-induced suppression of Prx weakened H_2_O_2_-scavenging activity [94].

### 4.2. Regulation of PTEN by Prx II

ROS has been demonstrated to participate in insulin signaling [95,96]. The downstream signaling of the PI3K/Akt pathway was activated when the cell was stimulated with insulin, which triggers the activation of specific receptors, including the insulin receptor (IR) and insulin receptor substrate (IRS). The GLUT4 was employed from the intracellular pool to the membrane surface after the insulin-induced activation of the downstream cascade, permitting the entry of glucose into the cell [97]. PTEN and PTPs all antagonize the insulin signaling as they directly interact with PI3K and IR [98], and both consist of a cysteine residue in the active site that is highly susceptible to H_2_O_2_-induced oxidation.

Prxs are well-known as peroxide-scavenging enzymes having a high affinity for H_2_O_2_, with half-maximal activity <20 µm [99]. Also, Prx II has shown involvement in various cellular processes, such as hemoglobin stability, hippocampal synaptic plasticity, osteoclasts, and maintaining the stemness of embryonic stem cells [100,101,102].

**Figure 3:**
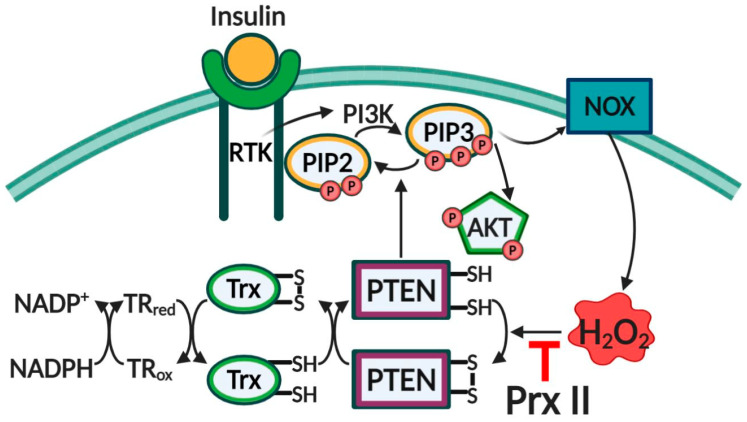
Role of Prx II in the redox regulation of PTEN. Insulin induced the activation of PI3K, resulting in the conversion of PIP2 to PIP3. PIP3 induced the production of H_2_O_2_ by activating the NOX complex. NOX-induced H_2_O_2_ oxidized PTEN and started the forming of the intramolecular disulfide bond, which inactivates PTEN lipid phosphatase. The presence of Prx II reduced PTEN oxidation levels. Black arrows (activating), red arrows (blocking).

It has been demonstrated that in the Prx II-deficient MEFs treated with insulin, the downstream signaling of the PI3K/Akt pathway increased, which was accompanied by an increase in oxidized PTEN levels and ROS levels [103]. After insulin treatment (100 nM) of MEFs, the levels of phosphorylated insulin receptor β (IRβ) were almost 1.6-fold higher in Prx II^−/−^ compared to in Wild type (WT) at 5 min [103]. In addition, the levels of PI3K and phosphorylated Akt in the Prx II-deficient MEFs were also higher than that of WT with 1.8-fold and 2.2-fold, respectively. Interestingly, PTP1B and PTEN oxidation levels in Prx II^−/−^ increased over time compared to that in WT MEFs. ROS levels were also increased in Prx II^−/−^ MEFs after exposure to insulin [103]. The participant of H_2_O_2_ in intracellular signaling by targeting PTEN and Prx II, and the regulation of H_2_O_2_ concentration by Prx II are depicted schematically in Figure 3.

### 4.3. Regulation of PTEN by Prx III

Lipoxygenases (LOX) are enzymes that catalyze the formation of hydroperoxyl-eicosatetraenoic acid (HpETE) from arachidonic acid (AA) and linoleic acid (LA), which could be important components in inflammatory and prooxidant mediators [104,105]. 15-Lipoxygenase (15-LOX) is a LOX family member that can promote the formation of a 15(s)-hydroperoxy-eicosatetraenoic acid (15s-HpETE) and a 15-hydroxyeicosatetraenoic acid (15s-HETE) from AA. There are two isoforms of human 15-LOX, called 15-LOX-1 and 15-LOX-2 [106,107]. 15s-HpETE is the main product after the catalysis of AA by 15-LOX-1. Small levels of 12s-HpETE are also synthesized in this catalysis. During the catalysis by 15-LOX-2, AA was metabolized mostly into 15s-HpETE but was not into 12s-HpETE [108]. The reduction and transformation of HpETEs then happened that causes the creation of eicosanoids, the vital lipid peroxides in the responsiveness of the immune system, and other physiological mechanisms. The increase of lipid peroxide levels is related to the pathological condition of differential human disorders and diseases, including neurodegeneration, atherosclerosis, type II diabetes, metabolic disorders, solid tumors, and hematologic malignancies, through the deterioration of biological oxidative processes [109,110,111,112].

**Figure 4:**
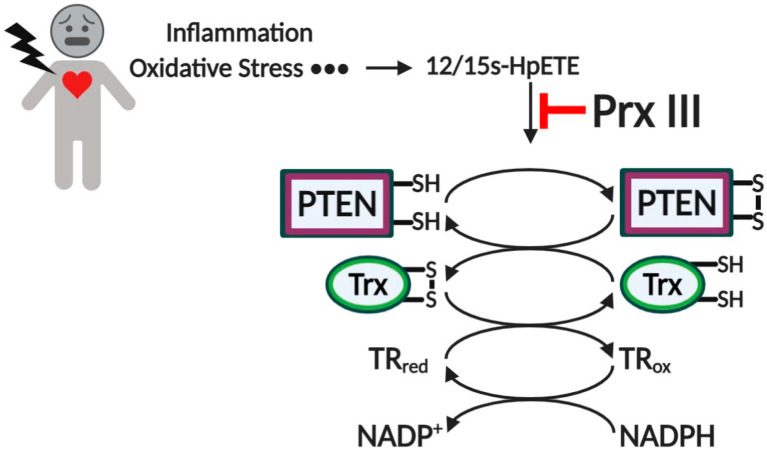
A schematic model for the effect of 12/15s-HpETE and Prx III on the redox regulation of PTEN. Prx III plays an important role in the control of endogenous lipid peroxide-induced redox regulation of PTEN. 12/15s-HpETE inhibits the Trx redox system by inducing dimerization of Trx, resulting in the delayed reduction of oxidized PTEN and oxidized Prx. Prx III prevents 12/15s-HpETE-mediated PTEN oxidation by catalyzing the reduction of lipid peroxide. Black arrows (activating), red arrows (blocking).

In MEFs, it was shown that both 15s-HpETE and 12s-HpETE induce the oxidation of PTEN and that oxidized PTEN was gradually reversed to the reduced form by cellular antioxidants after 30 min of exposure [113]. Similar disulfide bond formation between Cys^124^ and Cys^71^ was observed in both H_2_O_2_- and 15s-HpETE-treated MEFs [113]. Moreover, Prx III-deficiency showed a higher level of PTEN oxidation compared to that of WT. Furthermore, Trx dimers were maintained for 60 min in Prx III^−/−^ MEFs compared to 5 min in Prx III^+/+^ MEFs [113]. These data suggested that Prx III might play an important role in protecting PTEN and Trx system from oxidation by lipid peroxides [113], which is depicted in Figure 4.

## 5. Conclusions

PTEN is well-known for the negative regulatory function of the PI3K/Akt pathway by dephosphorylating PIP3 to PIP2. However, ROS, which can be produced as a secondary messenger of intracellular signaling, has the ability to induce PTEN oxidation, forming an intramolecular disulfide bond and inactivating PTEN phosphatase function. In the cells, Prxs, the scavengers for peroxides, plays a crucial role in the redox regulation of PTEN and maintaining redox homeostasis. The redox regulation of PTEN by Prxs has been recently investigated in some contexts. The main focus of this review is on the redox regulation of PTEN concerning Prxs.

Prx I has been proved to preserve PTEN under mild oxidative stress by directly interacting with PTEN. However, under the high concentrations of H_2_O_2_, Prx I was hyperoxidized and dissociated from PTEN. Besides, Prx II-deficient MEFs induced PTEN oxidation and increased PI3K/Akt activation when exposed to insulin, which leads to an increase the insulin sensitivity. Additionally, the deficiency of Prx II in Hela cells increased the PIP3 accumulation and Akt activation following the stimulation of growth factors [21]. Growth factor stimulation also induced PTEN oxidation in Hela cells [21]. Therefore, the cytosolic Prx II may function to protect PTEN from oxidation. However, it has also been reported that direct interaction between PTEN and Prx II was not observed by immunoprecipitation [93,114]. Further studies are necessary to investigate the relation between redox regulation of PTEN and Prx II. Prx III-deficiency also induces the augmentation in both PTEN oxidation and Trx dimerization. This provides a new line of evidence regarding the role of Prxs in the redox regulation of PTEN. As Prx III localizes in the mitochondria, it can be speculated that Prx III directly reduces peroxides radicals, which decreases 12/15s-HpETE levels and subsequently declines PTEN oxidation levels. However, further investigation focusing on the mechanism of Prx III in regulating PTEN redox status is also needed.
